# Statistical methods for the computation and parameter estimation of a fractional SIRC model with Salmonella infection

**DOI:** 10.1016/j.heliyon.2024.e30885

**Published:** 2024-05-13

**Authors:** Salem Mubarak Alzahrani

**Affiliations:** Mathematics Department, Faculty of Science, Al-Baha University, Saudi Arabia

**Keywords:** Stochastic approaches, Milstein method, Sensitivity analysis and parameter estimation, Salmonella bacterial infection, Statistical analysis

## Abstract

This study analyzes the fractional order SIRC epidemic model under stochastic fractional differential equations in the Caputo sense. This article describes Salmonella infection in animal herds. For the deterministic system, we explain solution positivity and boundness. We also prove that the fractional stochastic solution exists and is unique. Other criteria considered include non-negativity of solutions, local and global stability analyses, Hyers-Ulam stability analysis, and sensitivity analysis for the deterministic system. Furthermore, according to the truncated Ito-Taylor expansion, we apply a numerical method to solve the stochastic fractional SIRC epidemic model, namely the Milstein method, to solve the stochastic fractional SIRC epidemic model. A comparison of the approximation solution and the corresponding deterministic model for different sample paths shows the efficiency of the numerical method. In addition, graphs and error tables provide insight into numerical experiments' results. The stochastic nature of the model allows random fluctuations in Salmonella infection spread. By incorporating uncertainty into the model, we gain a more realistic understanding of the epidemic dynamics and can better evaluate the effectiveness of control measures. Additionally, the numerical method used to solve the stochastic fractional SIRC epidemic model provides valuable insights into the variability of the results. This enhances our ability to make informed decisions about managing and preventing bacterial infections in animal herds.

## 1 Introduction

Salmonella infection [1] is a common illness caused by Salmonella bacteria. This disease results in diarrhea, fever, and abdominal cramps. Among the most common causes of public health issues is Salmonella. Humans and animals are susceptible to Salmonella infection, which is a major zoonotic disease. It takes 12 to 72 hours after infection to experience diarrhea, fever, and abdominal cramps. After 4 to 7 days of illness, most people recover without treatment. Some patients may need hospitalization due to severe diarrhea. Humans and animals are infected with Salmonella in their intestinal tracts. Food infected with animal feces transmits Salmonella to humans. Normal-looking and smelling foods are usually contaminated. In addition to beef, poultry, milk, or eggs, contaminated foods may include any food, including vegetables. Mathematics are crucial to understanding Salmonella bacteria transmission in a particular environment. Additionally, it can help predict outbreaks. Mathematical analysis also leads to determining equilibrium states and suggesting actions for decision makers to control the disease's spread. Biological systems have largely been modeled using integer-order ordinary differential equations (or delay differential equations), see for example, [2], [3], [4]. SIR (Susceptible-Infected-Recovered) is the classical model that the authors propose in [1]. In Din et al. [5], stochastic dynamics were employed to account for variations in dengue fever spread. Based on multiple optimal control, Ndii and Adi found that awareness in malaria infection communities was the best way to reduce malaria transmission in [6]. Using a one-locus model, Suandi et al. [7] investigated how anopheles mosquitoes evolved resistance against insecticides. Using fractional stochastics, Alkahtani and Koca [8] determined the existence and uniqueness of solutions to an SIR model. Akinlar et al. [9] constructed a mathematical model incorporating white noise. Omar et al., [10] studied the COVID model as it applied to Egypt and found conclusive evidence that stochastic factors affected COVID spread. In Sweilam et al. [11] a stochastic fraction order COVID model was developed and its dynamics were investigated. Anangana and Araz [12] modeled COVID and presented a numerical scheme for solving fractional stochastic models. With stochastic approaches, they showed the COVID model solutions exist and are unique. Various studies have demonstrated that stochastic simulation of a model can provide information that is comparable to actual data [13]. There is much research in the literature on analyzing the effects of COVID-19 through mathematical modeling, see [14], [15], [16], [17]. In 2019, Tilahun et al. [18] investigated a mathematical model of pneumonia and bacterial meningitis. In 2019, Mbabazi et al. [19] provided a mathematical model of pneumococcal pneumonia with temporal delays, prompting an analysis of a Hopf-bifurcation. Otoo et al. [20] evaluated a model of bacterial pneumonia transmission. The investigation revealed how immunization prevents illness. In a graphic presentation, Zephaniah et al. [21] presented dynamic mathematical pneumonia models. Corona virus pneumonia is on the rise in Wuhan, China, as reported by Ming and colleagues [14]. Through a battery of clinical testing, Jung et al. [22] confirmed the findings and identified a previously unknown pathogen as the root of the sickness. In [23], Wafula et al. created a deterministic mathematical model of pneumonia-HIV co-infection, using anti-pneumonia and ART therapy treatments as controls to explain the optimal control, see also, [24], [25]. Oluwatobi et al. [26] looked at the impact of pneumonia, the presence of fundamental and effective reproduction numbers, and the stability of equilibrium points. Researchers have presented models for modeling pneumonia dynamics based on a review of the literature such as [27], [28], [29]. To better understand the factors that contribute to pneumonia spread, they developed a dynamic model. Muhammad Naveed et al. researched the delayed variant of the SCIR model and its mathematical analysis [30]. Several mathematicians have developed mathematical models on the interaction of glucose–insulin interaction based on IVGTT in the last several decades, which have been presented in the literature, see [31], [32], [33], [34], [35], [36], [37]. Epidemiology is crucial in several disciplines, including medicine, engineering, chemistry, physics, economics, and many more, see [38], [39], [40], [41]. To examine a disease model, researchers use spatial-temporal, stochastic, fractional, and fractal mathematics, see [42], [43], [44], [45]. Several mathematicians have developed mathematical models on the fractional-order SIRC model in the last several decades, which have been presented in the literature, see [46], [47], [48], [49].

Statistical models are critical for many reasons. Fractional-order models can more accurately represent certain phenomena, such as complex biological or physical systems. They also provide a simpler and more intuitive way of understanding such systems' behavior. Fractional-order models can also solve complex mathematical problems. Traditional models ignore the details of the epidemic being studied, making nonlocal operators the only choice for incorporating memory effects. Recent scientific literature has seen the proposal of multiple epidemic models for the virus spread. The most popular is called Caputo, and it is the most appropriate choice to replace the integer-order time derivative in the proposed model. Caputo's revision of traditional epidemiological models is better than the standard (classical: integer-order derivatives) one. Epidemiological research published recently demonstrated that older models failed to account for the complex dynamics of infection spread. Using a fractional-order operator for any physical or biological model, one can verify that the fractional parameter has no physical or geometrical meaning. The SIRC model, a cross-immune compartment, was introduced by the authors in [50]. The newly introduced compartment cross-immune C describes a state between fully susceptible S and fully protected R, see [51]. There has been recent discussion of the fractional SIRC model for influenza, a disease affecting humans. Deterministic predictions are difficult for diseases such as Salmonella bacteria infections. When modeling Salmonella bacterial infection transmission, fluctuation should be taken into account since the infection rate can change at any time.

In this work, fractional stochastics is applied to account for randomness behavior in the Salmonella infection model. The fractional stochastic model will provide more detailed information about the proposed model than the deterministic model. This is due to crossover and random properties. This study will examine the existence and uniqueness of solutions to the proposed model. A deterministic model solution is non-negative and bounded. By determining $R_{0}$, we can determine how the system will behave dynamically. A sufficient number of conditions are also met for the model to be stable or unstable. Under some conditions, equilibrium can be derived globally. Furthermore, Hyers-Ulam stability has been established. This study employs stochastic fractional differential equations to analyze and simulate Salmonella bacterial infection in animal herds. Based on the nonlinear system shown below, this study constructs a generalized epizootic framework for Salmonella bacterial infection. Using a stochastic threshold technique would reduce pandemic risk, according to the system's research. Using stationary distributions and ergodicity, we systematically assess the stochastic framework for virus eradication. The numerical investigation of the proposed system is evaluated by considering the Caputo formulation featuring white noise. To bolster our theoretical findings, we provide simulated outcomes. We studied the Milstein scheme based on the truncated Ito-Taylor expansion proposed in [52]. This scheme was then applied to a nonlinear fractional order SIRC epidemic model to demonstrate its efficiency.

There are several stochastic factors involved in Salmonella infection transmission. This study developed a stochastic Salmonella epidemic model with noise influence. The stochastic effects of climate factors, immigration, and human behavior were considered. The problem is well behaved in terms of some of its fundamental properties, such as boundedness and the fact that there is a unique positive global solution to the system (2.2), which shows that the problem is well-behaved. In addition, we demonstrated that the stochastic model (2.2) has a unique stationary distribution whenever the white noise intensity and reproduction number are sufficiently small. As a final step, computer generated solutions are also provided to verify our theoretical findings. Based on the results of this study, it appears that there should be a lot of effort made in screening under appropriate guidance so that this disease can be prevented. Furthermore, the approach presented here may also be applicable when analyzing other epidemics such as tuberculosis, malaria, hepatitis B, and the current COVID-19 pandemic, etc., by simply modifying the approach presented here.

The limitation of the proposed method lies in the use of fractional calculus to model the epidemic. Fractional calculus introduces a non-integer order derivative to the model, which does not necessarily correspond to a physical interpretation of the epidemic dynamics. The traditional SIR (Susceptible-Infectious-Recovered) model, and its extensions, provide a robust and widely accepted framework for modeling infectious diseases. In contrast, fractional calculus is still a relatively novel approach in epidemiology. While it has its applications, its use in this context might have limitations due to its theoretical nature. Though the fractional SIRC (Susceptible-Infectious-Recovered-Carrier) model might produce meaningful results, its validity in capturing the intricacies of an epidemic could be limited. It's crucial to consider that the use of fractional calculus in epidemic modeling is an active area of research, and the full extent of its applicability and limitations in this context is not yet fully understood. Therefore, caution should be exercised when interpreting the results produced by this method.

## 2 Stochastic model formulation

**Figure 1:**
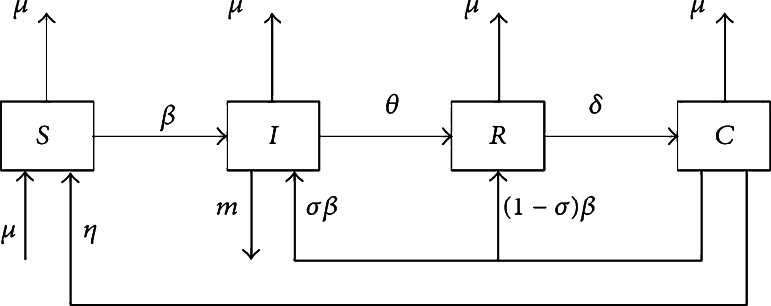
Schematic diagram of SIRC model [51].

Suppose Salmonella infection spreads through herds grouped into four compartments according to their infection status: S indicates the proportion of susceptible individuals at time *t*. It is determined that I is the proportion of infected individuals (those who have contracted the bacterial infection), R is the proportion of individuals who have recovered from infection and are temporarily immune, and C is the proportion of individuals who have acquired cross immunity as of time *t*. The total number of animals in a herd can be calculated using the formula N=S+I+R+C. All animals are susceptible to infection. If a susceptible individual becomes infected, the infectious individual leaves the susceptible compartment and enters the infectious compartment, where the infectious individual becomes infected. Infected animals should be placed in recovery areas as soon as possible. It is reported that individuals who receive chemotherapy are temporary immune recipients. They are grouped in the compartment C for a period of time. The disease transmission model can be seen as a system of equations in which nonnegative initial conditions along with an equation system are used to describe the disease transmission process. Introducing fractional order into the model (2.1), we assume that S=S/N, I=I/N,R=R/N, and C=C/N, where N is the total population. SIRC models based on classical differential operators have no memory or heredity. We can examine the classical SIRC model with fractional derivatives when considering heredity and memory. These effects can only be considered in fractional cases. Due to this, the model looks like this as shown in [51]:

(2.1) Dtυ0CS=μ−(μ+βI)S+ηC,Dtυ0CI=σβIC+βIS−(m+μ+θ)I,Dtυ0CR=θI+β(1−σ)IC−(δ+μ)R,Dtυ0CC=−βIC−(μ+η)C+δR,

with $S(0)=S_{0},I(0)=I_{0}$, $R(0)=R_{0}$, and $C(0)=C_{0}$. *μ* represents the mortality rate in each compartment and is assumed to be equal to the birth rate. *β* is also known as the transmission rate from susceptible to infected. $\eta^{-1}$ is the crossimmune period, $\theta^{-1}$ is the infectious period, $\delta^{-1}$ is the total immune period, and *σ* represents the fraction of crossimmunes recruited into the infective subpopulation. Fig. 1 shows that the disease also induces a mortality rate of *m*.

The main difference between the SIR model and the SIRC model lies in the inclusion of an additional compartment for cross-immunity in the SIRC model. In the SIRC model, an additional compartment for cross-immunity (C) is introduced. This compartment represents individuals who have acquired immunity to the infection due to cross-immunity, meaning they may have been exposed to a similar pathogen in the past or have been vaccinated against it. Individuals in this compartment can potentially move back to the susceptible compartment if their immunity wanes over time, but they can also move back to the susceptible compartment due to the loss of immunity.

In order to extend the fractional model (2.1) so that it can include stochastic terms in the fractional case, the equation needs to be reformulated so that it will include the stochastic compartment as follows:

(2.2) Dtυ0CS=μ−(μ+βI)S+ηC+σ1G1(S,t)W˙1,Dtυ0CI=σβIC+βIS−(m+μ+θ)I+σ2G2(I,t)W˙2,Dtυ0CR=θI+β(1−σ)IC−(δ+μ)R+σ3G3(R,t)W˙3,Dtυ0CC=−βIC−(μ+η)C+δR+σ4G4(C,t)W˙4.

There are Brownian motions in $W_{i},(i=1,2,3,4)$. Specifically, $\sigma_{i}$, (i=1,2,3,4) represents the intensity of noise, while $G_{1}(S,t)$, $G_{2}(I,t)$, $G_{3}(R,t)$ and $G_{4}(C,t)$ are stochastic constants. A diffusion coefficient associated with each of the variables in the extended model can be defined as $\sigma_{1},\sigma_{2},\sigma_{3}$ and $\sigma_{4}$.

Definition 1 Considering a function defined as ϕ:[a,b]→R, the fractional derivative of order *p* (left) is expressed in the following manner:

DtυaCϕ=1Γ(n−τ)∫atϕ(n)(ϵ)(t−ϵ)n−τ−1dϵ,n−1<τ≤n.

Definition 2 Fractional Caputo's integral operator is defined as

(2.3) ItυaCϕ=1Γ(υ)∫atϕ(ϵ)(t−ϵ)υ−1dϵ.

Definition 3 **[53], [54]**

Stochastic processes consist of random variables {ϕ(x)|x≥0}.

Definition 4 **[53], [54], Levy process**

Take a stochastic process {ϕ(x)|x≥0} with a complete probability space (Θ,M,P). Levy processes are classified as *ϕ* if

(i) It is almost certain that *ϕ* meets the condition ϕ(0)=0,
(ii) The increments are independent and stationary, and
(iii) For b>0 and c>0, since it is stochastically continuous, we can say that
  $$\lim_{t\to c}P_{r}(|\phi (x)-u(c)|>b)=0.$$

Definition 5 **[53], [54], Chebyshev's inequality**

If *u* is a random variable and τ∈[1,∞) is a constant, then

$$P_{r}\left(|\phi |\mu^{-1}\geq 1\right)\leq E_{x}\left(|\phi {|}^{\upsilon }\right)\mu^{-\tau },\;\text{ for all }\;\mu >0.$$

Definition 6 **[53], [54], Borel Cantelli lemma**

If $\zeta_{k}\subset M$ and $\sum_{k=1}^{\infty }P_{r}\left(\zeta_{k}\right)<\infty$, then

$$P_{r}\left(\lim_{k\to \infty }\sup\zeta_{k}\right)=0.$$

Theorem 1 **[53], [54]**

(i) *If* ${\phi_{m}|}_{m=1}^{\infty }$ *is a submartingale, then*
  $$P_{r}\left(\mu^{-1}\max_{1\leq k\leq m}\phi_{k}\geq 1\right)\leq E_{x}\left(\phi_{m}^{+}\right),$$
  μ>0 *and* ∀m=1,2,… *.*
(ii) *If* 1<p<∞ *and* ${\phi_{m}|}_{m=1}^{\infty }$ *is a martingale, then*
  $$E_{u}\left(\max_{1\leq k\leq m}{\left|\phi_{k}\right|}^{\upsilon }\right)\leq p^{\upsilon }{\left(p-1\right)}^{-p}E_{x}\left({\left|\phi_{m}\right|}^{\upsilon }\right),\;\forall m=1,2,\ldots$$

Definition 7 **[53], [54]**

A real-time positive number process ${\left(W\right)}_{t\geq 0}$ is called a Wiener process if it is described as follows:

1. W(0)=0,P− a.s.
2. ∀0≤s<t,W−W(s) are independent of $M_{s}$.
3. ∀0≤s<t,W−W(s)∽N(0,t−s).

There are three basic forms of n-dimensional stochastic differential equations (SDEs), which are defined as follows:

$$dY=F(t,Y)dt+G(t,Y)\overset{˙}{W},\text{ with }y_{0}=Y\left(0\right).$$

There are two types of Wiener processes: the standard Wiener process W, which is an n-dimensional Wiener process with drift coefficients *F* and diffusion coefficients *G*.

Theorem 2 *A unique solution exists (*S*,* I*,* R*,* C*) to* $E_{q}$ *for any initial value* $(S(0),I(0),R(0),C(0))\in R_{+}^{4}$*. In most cases, the solution* $(S(0),I(0),R(0),C(0))\in R_{+}^{4}$ *will remain in* $R_{+}^{4}$ *with probability one for all* t≥0 *almost certainly.*

## 3 Stochastic approaches

### 3.1 Ito-Taylor expansion

Following [52], we discuss the Ito-Taylor expansion. Numerical analysis relies heavily on the Taylor formula. With the Taylor formula, we can obtain an approximation to any desired order of accuracy of a sufficiently smooth function in a given neighborhood. Stochastic expansion formulas corresponding to the deterministic Taylor formula are beneficial for extending the increments of smooth functions of ITo processes. There are some possibilities with such a stochastic Taylor formula. The Ito-Taylor expansion is one possible method of getting this expansion. Assume that the representation of a stochastic differentiable function is as follows: $dW_{i}=W_{i}\;dt$. For the stochastic case, we can obtain an Ito-Taylor expansion. Consider

(3.1) dY=F(Y)dt+g(Y)dW.

In which *f* and *g* satisfy a linear growth bound and are sufficiently smooth.

From Ito's lemma, let F be a twice continuously differentiable function of Y

(3.2) $$\begin{array}{cc} dF[Y]= & \left\{F[Y]\frac{\partial F[Y]}{\partial Y}+\frac{1}{2}g^{2}[Y]\frac{\partial^{2}F[Y]}{\partial Y^{2}}\right\}dt+g[Y]\frac{\partial F[Y]}{\partial Y}dW. \end{array}$$

Following is a definition of the operators:

(3.3) $$L^{0}\equiv F[Y]\frac{\partial }{\partial Y}+\frac{1}{2}g^{2}[Y]\frac{\partial^{2}}{\partial Y^{2}},$$

(3.4) $$L^{1}\equiv g[Y]\frac{\partial }{\partial Y},$$

(3.2) becomes

(3.5) $$dF[Y]=L^{0}F[Y]dt+L^{1}F[Y]d\;W,$$

and integral form of (3.5) is

(3.6) $$F[Y]=F\left[Y\left(t_{0}\right)\right]+\int_{t_{0}}^{t}L^{0}F[Y(\theta )]d\theta +\int_{t_{0}}^{t}L^{1}F[Y(\theta )]d\;W(\theta ).$$

Based on F(x)=x, F(x)=F(x), and F(x)=g(x), (3.2) becomes as follows:

(3.7) $$Y=Y\left(t_{0}\right)+\int_{t_{0}}^{t}F[Y(\theta )]d\theta +\int_{t_{0}}^{t}g[Y(\theta )]d\;W(\theta ),$$

(3.8) $$F[Y]=f\left[Y\left(t_{0}\right)\right]+\int_{t_{0}}^{t}L^{0}F[Y(\theta )]d\theta +\int_{t_{0}}^{t}L^{1}F[Y(\theta )]d\;W(\theta ),$$

(3.9) $$g[Y]=g\left[Y\left(t_{0}\right)\right]+\int_{t_{0}}^{t}L^{0}g[Y(\theta )]ds+\int_{t_{0}}^{t}L^{1}g[Y(\theta )]d\;W(\theta ).$$

Substituting (3.8) and (3.9) into (3.7), one obtains:

(3.10) $$\begin{array}{cc} Y= & Y\left(t_{0}\right)+\int_{t_{0}}^{t}(f\left[Y\left(t_{0}\right)\right]+\int_{t_{0}}^{\theta_{1}}L^{0}f\left[Y\left(\theta_{2}\right)\right]d\theta_{2}\\ & +\int_{t_{0}}^{\theta_{1}}L^{1}f\left[Y\left(\theta_{2}\right)\right]d\;W\;\left(\theta_{2}\right))d\theta_{1}\\ & +\int_{t_{0}}^{t}(g\left[Y\left(t_{0}\right)\right]+\int_{t_{0}}^{\theta_{1}}L^{0}g\left[Y\left(\theta_{2}\right)\right]d\theta_{2}\\ & +\int_{t_{0}}^{\theta_{1}}L^{1}g\left[Y\left(\theta_{2}\right)\right]d\;W\;\left(\theta_{2}\right))d\;W\;\left(\theta_{1}\right), \end{array}$$

and therefore,

(3.11) $$Y=Y\left(t_{0}\right)+f\left[Y\left(t_{0}\right)\right]\int_{t_{0}}^{t}d\theta_{1}+g\left[Y\left(t_{0}\right)\right]\int_{t_{0}}^{t}d\;W\;\left(\theta_{1}\right)+R.$$

Taking into account the double integral terms, R is the remaining term.

(3.12) $$\begin{array}{cc} R\equiv & \int_{t_{0}}^{t}\int_{t_{0}}^{\theta_{1}}L^{0}f\left[Y\left(\theta_{2}\right)\right]d\theta_{2}d\theta_{1}+\int_{t_{0}}^{t}\int_{t_{0}}^{\theta_{1}}L^{1}f\left[Y\left(\theta_{2}\right)\right]d\;W\;\left(\theta_{2}\right)d\theta_{1}\\ & +\int_{t_{0}}^{t}\int_{t_{0}}^{\theta_{1}}L^{0}g\left[Y\left(\theta_{2}\right)\right]d\theta_{2}d\;W\;\left(\theta_{1}\right)+\int_{t_{0}}^{t}\int_{t_{0}}^{\theta_{1}}L^{1}g\left[Y\left(\theta_{2}\right)\right]d\;W\;\left(\theta_{2}\right)d\;W\;\left(\theta_{1}\right). \end{array}$$

When we select $F=L^{1}g$ in (3.6), we obtain

(3.13) $$\begin{array}{cc} & \int_{t_{0}}^{t}\int_{t_{0}}^{\theta_{1}}L^{1}g\left[Y\left(\theta_{2}\right)\right]d\;W\;\left(\theta_{2}\right)d\;W\;\left(\theta_{1}\right)=\int_{t_{0}}^{t}\int_{t_{0}}^{\theta_{1}}(L^{1}g\left[Y\left(t_{0}\right)\right]\\ & \;+\int_{t_{0}}^{\theta_{2}}L^{0}L^{1}g\left[Y\left(\theta_{3}\right)\right]d\theta_{3}+\int_{t_{0}}^{\theta_{2}}L^{1}L^{1}g\left[Y\left(\theta_{3}\right)\right]d\;W\;\left(\theta_{3}\right))d\;W\;\left(\theta_{2}\right)d\;W\;\left(\theta_{1}\right), \end{array}$$

and using $L^{1}g=gg^{\prime }$, yields

(3.14) $$\begin{array}{cc} Y= & Y\left(t_{0}\right)+f\left[Y\left(t_{0}\right)\right]\int_{t_{0}}^{t}d\theta_{1}+g\left[Y\left(t_{0}\right)\right]\int_{t_{0}}^{t}d\;W\;\left(\theta_{1}\right)\\ & +g\left[Y\left(t_{0}\right)\right]g^{\prime }\left[Y\left(t_{0}\right)\right]\int_{t_{0}}^{t}\int_{t_{0}}^{\theta_{1}}d\;W\;\left(\theta_{2}\right)d\;W\;\left(\theta_{1}\right)+\overset{˜}{R}. \end{array}$$

We now have a new remainder $\overset{˜}{R}$:

(3.15) $$\overset{˜}{R}\equiv \int_{t_{0}}^{t}\int_{t_{0}}^{\theta_{1}}L^{0}f\left[Y\left(\theta_{2}\right)\right]d\theta_{2}d\theta_{1}+\int_{t_{0}}^{t}\int_{t_{0}}^{\theta_{1}}L^{1}f\left[Y\left(\theta_{2}\right)\right]d\;W\;\left(\theta_{2}\right)d\theta_{1}+\int_{t_{0}}^{t}\int_{t_{0}}^{\theta_{1}}L^{0}g\left[Y\left(\theta_{2}\right)\right]d\theta_{2}d\;W\;\left(\theta_{1}\right)+\int_{t_{0}}^{t}\int_{t_{0}}^{\theta_{1}}\int_{t_{0}}^{\theta_{2}}L^{0}L^{1}g\left[Y\left(\theta_{3}\right)\right]d\left(\theta_{3}\right)d\;W\;\left(\theta_{2}\right)d\;W\;\left(\theta_{1}\right)+\int_{t_{0}}^{t}\int_{t_{0}}^{\theta_{1}}\int_{t_{0}}^{\theta_{2}}L^{1}L^{1}g\left[Y\left(\theta_{3}\right)\right]d\;W\;\left(\theta_{3}\right)d\;W\;\left(\theta_{2}\right)d\;W\;\left(\theta_{1}\right).$$

Thus, the Ito-Taylor expansion for process (3.1) as (3.14) is obtained. As a result of applying Ito's lemma once again, we have

(3.16) $$\int_{t_{0}}^{t}\int_{t_{0}}^{\theta_{1}}d\;W\;\left(\theta_{2}\right)d\;W\;\left(\theta_{1}\right)=\frac{1}{2}{\left[W-W\;\left(t_{0}\right)\right]}^{2}-\frac{1}{2}\left(t-t_{0}\right).$$

Stochastic Taylor expansion obtained by writing (3.16) into (3.14).

(3.17) $$\begin{array}{cc} Y= & Y\left(t_{0}\right)+f\left[Y\left(t_{0}\right)\right]\int_{t_{0}}^{t}d\theta_{1}+g\left[Y\left(t_{0}\right)\right]\int_{t_{0}}^{t}d\;W\;\left(\theta_{1}\right)\\ & +g\left[Y\left(t_{0}\right)\right]g^{\prime }\left[Y\left(t_{0}\right)\right]\left\{\frac{1}{2}{\left[W-W\;\left(t_{0}\right)\right]}^{2}-\frac{1}{2}\left(t-t_{0}\right)\right\}+\overset{˜}{R}. \end{array}$$

As a consequence, using the Ito-Taylor expansion (3.17), the numerical integration scheme for the SDE can be formulated with a time discretization $0=t_{0}<t_{1}<\cdots <t_{n}<\cdots <t_{N}=T$ of a time interval [0,T] as follows:

(3.18) $$\begin{array}{cc} Y\left(t_{i+1}\right) & =Y\left(t_{i}\right)+f\left(Y\left(t_{i}\right)\right)\Delta t+g\left(Y\left(t_{i}\right)\right)\Delta W_{i}\\ & +\frac{1}{2}g\left(Y\left(t_{i}\right)\right)g^{\prime }\left(Y\left(t_{i}\right)\right)\left[{\left(\Delta W_{i}\right)}^{2}-\Delta t\right]+\overset{˜}{R}, \end{array}$$

where $\Delta t=t_{i+1}-t_{i}$ and $\Delta W_{i}=W\;\left(t_{i+1}\right)-W\;\left(t_{i}\right)$ for I=0,1,2,…,N−1 with the initial condition $Y\left(t_{0}\right)=Y_{0}$. The random variables $\Delta W_{i}$ are independent N(0,Δt) normally distributed random variables.

### 3.2 Milstein method

Milstein method can be obtained by truncating stochastic Taylor series after second order terms as follows:

$$Y\left(t_{i+1}\right)=Y\left(t_{i}\right)+f\left(Y\left(t_{i}\right)\right)\Delta t+g\left(Y\left(t_{i}\right)\right)\Delta W_{i}+\frac{1}{2}g\left(Y\left(t_{i}\right)\right)g^{\prime }\left(Y\left(t_{i}\right)\right)\left[{\left(\Delta W_{i}\right)}^{2}-\Delta t\right],$$

where *f* and *g* are twice continuously differentiable, and $f,f^{\prime },g,g^{\prime }$ are uniform Lipschitz functions. Take $Y\left(t_{0}\right)=Y_{0}$ with i=0,1,2,…,N−1. Milstein's approximation converges with strong order 1.0 under the $E[{\left(Y\left(0\right)\right]}^{2}<\infty$ assumption. In the case of additive noise, SDE is the differentiation of $f,f^{\prime },g,g^{\prime }$. Based on Milstein's numerical approximation method for the SDE [55], we simulate the dynamics of model (2.2). In this case, the discrete system (2.2) can be written as follows:

$$\left\{ \begin{array}{cc} S_{i+1} & =S_{i}+\left(\mu +\eta C_{i}-(\beta I_{i}+\mu )S_{i}\right)\Delta t+\sigma_{1}S_{i}\;\sqrt{\Delta t}W_{1,i}\\ & +\frac{\sigma_{1}}{2}S_{i}\;\left(W_{1,i}^{2}-1\right)\Delta t,\\ I_{i+1} & =I_{i}+\left(\beta S_{i}\;I_{i}+\sigma \beta C_{i}\;I_{i}-(m+\mu +\theta )I_{i}\right)\Delta t+\sigma_{2}I_{i}\;\sqrt{\Delta t}W_{2,i}\\ & +\frac{\sigma_{3}}{2}I_{i}\;\left(W_{2,i}^{2}-1\right)\Delta t,\\ R_{i+1} & =R_{i}+\left(\beta (1-\sigma )C_{i}\;I_{i}+\theta I_{i}-(\delta +\mu )R_{i}\right)\Delta t+\sigma_{3}R_{i}\;\sqrt{\Delta t}W_{3,i}\\ & +\frac{\sigma_{5}}{2}R_{i}\;\left(W_{3,i}^{2}-1\right)\Delta t,\\ C_{i+1} & =C_{i}+\left(\delta R_{i}-\beta C_{i}\;I_{i}-(\mu +\eta )C_{i}\right)\Delta t+\sigma_{4}{\overset{˜}{b}}_{i}\sqrt{\Delta t}W_{4,i}\\ & +\frac{\sigma_{4}}{2}{\overset{˜}{b}}_{i}\left(W_{4,i}^{2}-1\right)\Delta t. \end{array}\right.$$

## 4 Existence and uniqueness of solutions in a stochastic model

Theorem 3 *Let* $q,\overset{¯}{q}$ *be a certain positive constant satisfies:*

(i) ∀∈{1,…,4}
  $${\left|\Psi (\phi_{1},t)-\Psi \left(\phi_{2},t\right)\right|}^{2}\leq q\left|\phi_{1}-\phi_{2}\right|.$$
(ii) ∀(ϕ,t)∈R×[0,T]
  $$|\Psi (\phi ,t){|}^{2}\leq \overset{¯}{q}\left(1+|\phi {|}^{2}\right).$$

Proof The system (2.2) can be rewritten as:

$$S=S(0)+\int_{0}^{t}F_{1}(\theta ,S)d\theta +\int_{0}^{t}\sigma_{1}G_{1}(\theta ,S){\overset{˙}{W}}_{1}(\theta ),I=I(0)+\int_{0}^{t}F_{2}(\theta ,I)d\theta +\int_{0}^{t}\sigma_{2}G_{2}(\theta ,I){\overset{˙}{W}}_{2}(\theta ),R=R(0)+\int_{0}^{t}F_{3}(\theta ,R)d\theta +\int_{0}^{t}\sigma_{3}G_{3}(\theta ,R){\overset{˙}{W}}_{3}(\theta ),C=C(0)+\int_{0}^{t}F_{4}(\theta ,C)d\theta +\int_{0}^{t}\sigma_{4}G_{4}(\theta ,C){\overset{˙}{W}}_{4}(\theta ).$$

For t∈[0,T], we need to have

$${\left|F_{i}\;\left(t,y_{i}\right)\right|}^{2}<k_{i}\left(1+{\left|y_{i}\right|}^{2}\right),{\left|G_{i}\;\left(t,y_{i}\right)\right|}^{2}<k_{i}\left(1+{\left|y_{i}\right|}^{2}\right),$$

and

$${\left|F_{i}\;\left(t,y_{i}^{1}\right)-F_{i}\;\left(t,y_{i}^{2}\right)\right|}^{2},<{\overset{¯}{k}}_{i}\left|y_{i}^{1}-y_{i}^{2}\right|,{\left|G_{i}\;\left(t,y_{i}^{1}\right)-G_{i}\;\left(t,y_{i}^{2}\right)\right|}^{2}<{\overset{¯}{k}}_{i}\left|y_{i}^{1}-y_{i}^{2}\right|,$$

where ${\left(F_{i}\;\left(t,y_{i}\right)\right)}_{i\in \{1,2,3,4\}},{\left(G_{i}\;\left(t,y_{i}\right)\right)}_{i\in \{1,2,3,4\}}$. For t∈[0,T], we obtain

$${\left|F_{1}(t,S)\right|}^{2}={\left|\mu +\eta C-(\beta I+\mu )S\right|}^{2}\leq 4\left(\mu^{2}+\eta^{2}|C\;{|}^{2}+\beta^{2}|I\;{|}^{2}|S\;{|}^{2}+\mu^{2}|S\;{|}^{2}\right)\leq 4\left(\mu^{2}+\eta^{2}\underset{0\leq t\leq T}{\sup}|C\;{|}^{2}+\beta^{2}\underset{0\leq t\leq T}{\sup}|I\;{|}^{2}|S\;{|}^{2}+\mu^{2}|S\;{|}^{2}\right)\leq 4\left(\mu^{2}+\eta^{2}{\left\|C^{2}\right\|}_{\infty }+\beta^{2}{\left\|I^{2}\right\|}_{\infty }|S\;{|}^{2}+\mu^{2}|S\;{|}^{2}\right)\leq 4\left(\mu^{2}+\eta^{2}{\left\|C^{2}\right\|}_{\infty }\right)\left(1+\frac{\beta^{2}{\left\|I^{2}\right\|}_{\infty }+\mu^{2}}{\mu^{2}+\eta^{2}{\left\|C^{2}\right\|}_{\infty }}|S\;{|}^{2}\right).$$

This yields

$${\left|F_{1}(t,S)\right|}^{2}\leq k_{1}\left(1+|S\;{|}^{2}\right),$$

with $\frac{\beta^{2}{\left\|I^{2}\right\|}_{\infty }+\mu^{2}}{\mu^{2}+\eta^{2}{\left\|C^{2}\right\|}_{\infty }}<1$. Similarly:

$${\left|F_{2}(t,E)\right|}^{2}\leq 3\left(\beta^{2}{\left\|S^{2}\right\|}_{\infty }|I\;{|}^{2}+\sigma^{2}\beta^{2}{\left\|C^{2}\right\|}_{\infty }|I\;{|}^{2}+{\left(m+\mu +\theta \right)}^{2}|I\;{|}^{2}\right)\leq k_{2}|I\;{|}^{2}\leq k_{2}\left(1+|I\;{|}^{2}\right).$$

Similarly, we get

$${\left|F_{3}(t,I)\right|}^{2}\leq 2{\left\|I^{2}\right\|}_{\infty }\left({\left(1-\sigma \right)}^{2}\beta^{2}{\left\|C^{2}\right\|}_{\infty }+\theta^{2}\right)\left(1+\frac{{\left(\delta +\mu \right)}^{2}}{{\left\|I^{2}\right\|}_{\infty }\left({\left(1-\sigma \right)}^{2}\beta^{2}{\left\|C^{2}\right\|}_{\infty }+\theta^{2}\right)}|R\;{|}^{2}\right)\leq k_{3}\left(1+|R\;{|}^{2}\right),$$

under the condition $\frac{{\left(\delta +\mu \right)}^{2}}{{\left\|I^{2}\right\|}_{\infty }\left({\left(1-\sigma \right)}^{2}\beta^{2}{\left\|C^{2}\right\|}_{\infty }+\theta^{2}\right)}<1$,

$${\left|F_{4}(t,R)\right|}^{2}\leq 3\left(\delta^{2}{\left\|R^{2}\right\|}_{\infty }+\beta^{2}{\left\|I^{2}\right\|}_{\infty }|C\;{|}^{2}+{\left(\mu +\eta \right)}^{2}|C\;{|}^{2}\right)\leq 3\delta^{2}{\left\|R^{2}\right\|}_{\infty }\left(1+\frac{\beta^{2}{\left\|I^{2}\right\|}_{\infty }+{\left(\mu +\eta \right)}^{2}}{\delta^{2}{\left\|R^{2}\right\|}_{\infty }}|C\;{|}^{2}\right)\leq k_{4}\left(1+|R\;{|}^{2}\right),$$

under the condition $\frac{\beta^{2}{\left\|I^{2}\right\|}_{\infty }+{\left(\mu +\eta \right)}^{2}}{\delta^{2}{\left\|R^{2}\right\|}_{\infty }}<1$. For t∈[0,T], one obtains

$${\left|G_{1}(t,S)\right|}^{2}\leq \sigma_{1}^{2}\left(1+|S\;{|}^{2}\right),{\left|G_{2}(t,I)\right|}^{2}\leq \sigma_{2}^{2}\left(1+|I\;{|}^{2}\right),{\left|G_{3}(t,R)\right|}^{2}\leq \sigma_{3}^{2}\left(1+|R\;{|}^{2}\right),{\left|G_{4}(t,C)\right|}^{2}\leq \sigma_{4}^{2}\left(1+|C\;{|}^{2}\right).$$

In the following manner, the Lipschitz condition for ${\left(F_{i}\;\left(t,y_{i}\right)\right)}_{i\in \{1,2,3,4\}}$ and ${\left(G_{i}\;\left(t,y_{i}\right)\right)}_{i\in \{1,2,3,4\}}$ is verified

$${\left|F_{1}\left(t,S_{1}\right)-F_{1}\left(t,S_{2}\right)\right|}^{2}={\left|\beta I+\mu \right|}^{2}{\left|S_{1}-S_{2}\right|}^{2}\leq 2\left(\beta^{2}{\left\|I^{2}\right\|}_{\infty }+\mu^{2}\right){\left|S_{1}-S_{2}\right|}^{2}\leq {\overset{¯}{k}}_{1}{\left|S_{1}-S_{2}\right|}^{2},{\left|F_{2}\left(t,I_{1}\right)-F_{2}\left(t,I_{2}\right)\right|}^{2}={\left|\beta S+\sigma \beta C-(m+\mu +\theta )\right|}^{2}{\left|I_{1}-I_{2}\right|}^{2}\leq 2\left(\beta^{2}{\left\|S^{2}\right\|}_{\infty }+\sigma^{2}\beta^{2}{\left\|C^{2}\right\|}_{\infty }+{\left(m+\mu +\theta \right)}^{2}\right){\left|I_{1}-I_{2}\right|}^{2}\leq {\overset{¯}{k}}_{2}{\left|I_{1}-I_{2}\right|}^{2},{\left|F_{3}\left(t,R_{1}\right)-F_{3}\left(t,R_{2}\right)\right|}^{2}=|(\delta +\mu ){|}^{2}{\left|R_{1}-R_{2}\right|}^{2}\leq {\left(\delta +\mu \right)}^{2}{\left|R_{1}-R_{2}\right|}^{2}\leq {\overset{¯}{k}}_{3}{\left|R_{1}-R_{2}\right|}^{2},F_{4}\left(t,{\overset{˜}{c}}_{1}\right)-{F_{4}\left(t,{\overset{˜}{c}}_{2}\right)|}^{2}=|\beta I-(\mu +\eta ){|}^{2}{\left|{\overset{˜}{c}}_{1}-{\overset{˜}{c}}_{2}\right|}^{2}\leq 2\left(\beta^{2}{\left\|I^{2}\right\|}_{\infty }+{\left(\mu +\eta \right)}^{2}\right){\left|{\overset{˜}{c}}_{1}-{\overset{˜}{c}}_{2}\right|}^{2}\leq {\overset{¯}{k}}_{4}{\left|{\overset{˜}{c}}_{1}-{\overset{˜}{c}}_{2}\right|}^{2}.$$

Also, we have

$${\left|G_{1}\;\left(t,S_{1}\right)-G_{1}\;\left(t,S_{2}\right)\right|}^{2}\leq \frac{3}{2}\sigma_{1}^{2}{\left|S_{1}-S_{2}\right|}^{2}\leq {\overset{¯}{k}}_{1}{\left|S_{1}-S_{2}\right|}^{2},{\left|G_{2}\;\left(t,I_{1}\right)-G_{2}\;\left(t,I_{2}\right)\right|}^{2}\leq \frac{3}{2}\sigma_{2}^{2}{\left|I_{1}-I_{2}\right|}^{2}\leq {\overset{¯}{k}}_{2}{\left|I_{1}-I_{2}\right|}^{2},{\left|G_{3}\;\left(t,R_{1}\right)-G_{3}\;\left(t,R_{2}\right)\right|}^{2}\leq \frac{3}{2}\sigma_{3}^{2}{\left|R_{1}-R_{2}\right|}^{2}\leq {\overset{¯}{k}}_{3}{\left|R_{1}-R_{2}\right|}^{2},{\left|G_{4}\;\left(t,{\overset{˜}{c}}_{1}\right)-G_{4}\;\left(t,{\overset{˜}{c}}_{2}\right)\right|}^{2}\leq \frac{3}{2}\sigma_{4}^{2}{\left|{\overset{˜}{c}}_{1}-{\overset{˜}{c}}_{2}\right|}^{2}\leq {\overset{¯}{k}}_{4}{\left|{\overset{˜}{c}}_{1}-{\overset{˜}{c}}_{2}\right|}^{2}.$$

Thus, the condition on linear growth holds so that

$$\max\left\{\frac{\beta^{2}{\left\|I^{2}\right\|}_{\infty }+\mu^{2}}{\mu^{2}+\eta^{2}{\left\|C^{2}\right\|}_{\infty }},\frac{\beta^{2}{\left\|I^{2}\right\|}_{\infty }+\mu^{2}}{\mu^{2}+\eta^{2}{\left\|C^{2}\right\|}_{\infty }},\frac{\beta^{2}{\left\|I^{2}\right\|}_{\infty }+{\left(\mu +\eta \right)}^{2}}{\delta^{2}{\left\|R^{2}\right\|}_{\infty }}\right\}<1.$$

then the solution of (2.2) is unique. □

## 5 Sensitivity analysis and parameter estimation

To find the equilibrium in model (2.1), we have put the following equations Dtυ0CS=0CDtυI=0CDtυR=0CDtυC=0. Consider $E_{0}=(1,0,0,0)$. The positive endemic equilibrium point is given by $E^{⋆}=\left(S^{⋆},I^{⋆},R^{⋆},C^{⋆}\right)$, where

$$S^{⋆}=-\frac{\sigma \delta \theta I^{⋆}}{(\delta +\mu )(\mu +\eta )+(\mu +\delta \sigma )\beta I^{⋆}}+\frac{m+\mu +\theta }{\beta },R^{⋆}=\frac{\theta I^{⋆}\;\left(\beta I^{⋆}+\eta +\mu \right)}{(\delta +\mu )(\mu +\eta )+(\mu +\delta \sigma )\beta I^{⋆}},C^{⋆}=\frac{\theta \delta I^{⋆}}{(\delta +\mu )(\mu +\eta )+(\mu +\delta \sigma )\beta I^{⋆}},$$

where $I^{⋆}$ is the positive root of ${\overset{˜}{b}}_{1}I^{⋆2}+{\overset{˜}{b}}_{2}I^{⋆}+{\overset{˜}{b}}_{3}$, and

$${\overset{˜}{b}}_{1}=-\beta^{2}[\mu (\theta +\mu +\delta \sigma )+m(\mu +\delta \sigma )],{\overset{˜}{b}}_{2}=\beta [\eta \theta \delta +\beta \mu (\mu +\delta \sigma )-(m+\mu +\theta )\times [(\mu +\eta )(\delta +\mu )+\mu (\delta \sigma +\mu )]+\mu \sigma \delta \theta ],{\overset{˜}{b}}_{3}=\beta \mu (\delta +\mu )(\mu +\eta )\left[1-\left(\frac{m+\mu +\theta }{\beta }\right)\right].$$

Consider the following matrices:

$$F=\left(\begin{array}{cc} \frac{\partial F_{1}}{\partial I}\; & \frac{\partial F_{1}}{\partial R}\\ \frac{\partial F_{2}}{\partial I}\; & \frac{\partial F_{2}}{\partial R} \end{array}\right)=\left(\begin{array}{cc} \beta \; & 0\\ 0\; & 0 \end{array}\right),V=\left(\begin{array}{cc} \frac{\partial V_{1}}{\partial I}\; & \frac{\partial V_{1}}{\partial R}\\ \frac{\partial V_{2}}{\partial I}\; & \frac{\partial V_{2}}{\partial R} \end{array}\right)=\left(\begin{array}{cc} m+\mu +\theta \; & 0\\ -\theta \; & \mu +\delta \end{array}\right).$$

Then we have the following:

$$FV^{-1}=\left[\begin{array}{cc} \beta \; & 0\\ 0\; & 0 \end{array}\right]\left[\begin{array}{cc} \frac{1}{\left(m+\mu +\theta \right)}\; & 0\\ 0\; & \frac{1}{\left(\delta +\mu \right)} \end{array}\right].$$

From above, at $E_{0}$, one obtains

$$R_{0}=\frac{\beta }{m+\mu +\theta }.$$

According to the parameters of our model, the following equation yields the sensitivity index of $R_{0}$:

(5.1) $$\Gamma_{\omega }^{R_{0}}=\frac{\partial R_{0}}{\partial \omega }\times \frac{\omega }{R_{0}},$$

where *ω* is a value from Table 1. Table 1 lists the sensitivity indices of $R_{0}$. It is easy to verify that

$$R_{0}=\frac{\beta }{m+\mu +\theta }=0.7075,\frac{\partial R_{0}}{\partial \beta }=\frac{1}{m+\mu +\theta }=\frac{1}{0.212}=4.7169811,\frac{\partial R_{0}}{\partial \theta }=\frac{\partial R_{0}}{\partial m}=\frac{\partial R_{0}}{\partial \mu }=\frac{-\beta }{{\left(m+\mu +\theta \right)}^{2}}=4.818,$$

implies

**Table 1:** Parameter values [51].

Parameter	Description	Value	Reference
*μ*	Rates of replacement and exit ( day ^−1^)	0.011	[49]
*β*	Susceptible to be infected contact rate ( animal ^−1^ day ^−1^)	0.15	[49]
*θ*	Rate of recovery of infected animals per day ^−1^	0.16	[51]
*m*	Mortality rate due to disease ( day ^−1^)	0.041	[51]
*η*	Period of cross-immunity	0.5	[49]
*σ*	Probability of reinfection on average of C	0.06	[51]
*δ*	Clusters appearing on average over time	1	[51]
*N*	Population total	345	[51]

Using (5.1), Table 2 displays the sensitivity index of each parameter in the model. Based on Table 2, the sensitivity indexes indicate that $R_{0}$ increases as *μ*, *β*, *θ*, *m* increase. Other parameters, on the other hand, have fixed values, so they cannot be changed. According to these indices, disease endemicity has increased in recent years based on the data provided. On the other hand, when the parameters *η*, *σ*, *δ* are decreased while the rest of the parameters are maintained, $R_{0}$ decreases when the parameters are decreased.

**Table 2:** The values of these parameters.

Parameter	*μ*	*β*	*θ*	*m*	*η*	*σ*	*δ*
Sensitivity Index	0.06698	1.000049	1.0895	0.24968	0	0	0

## 6 Model mean and confidence interval

**Figure 2:**
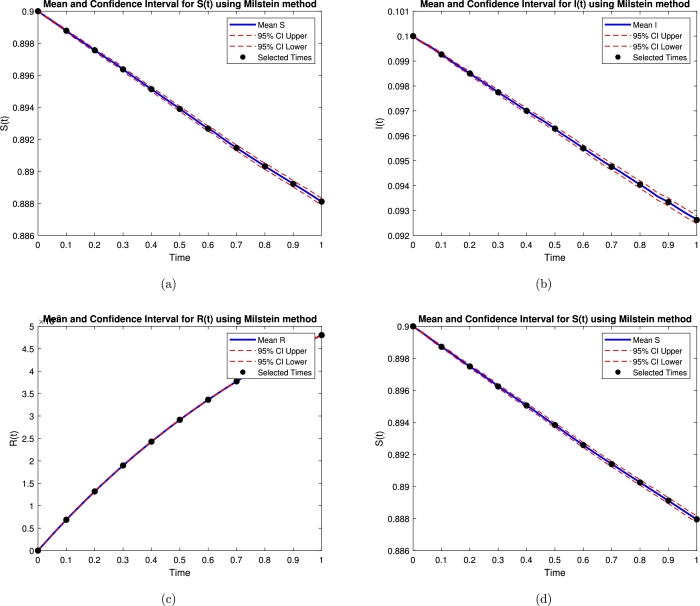
Mean and confidence interval for S, I, R, C.

**Figure 3:**
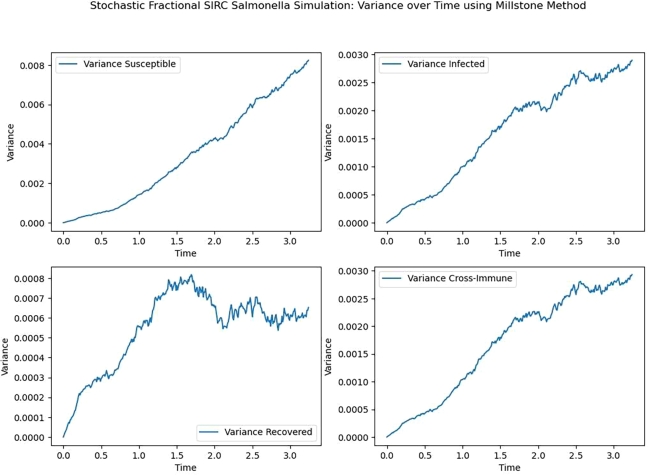
Variance for S, I, R, C.

**Figure 4:**
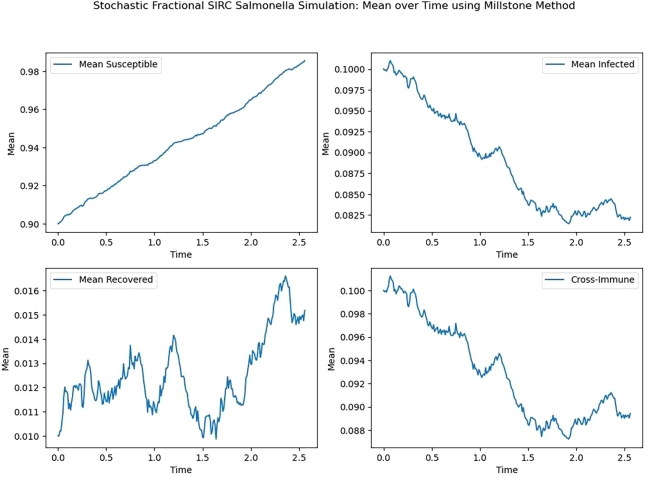
Mean for S, I, R, C.

**Figure 5:**
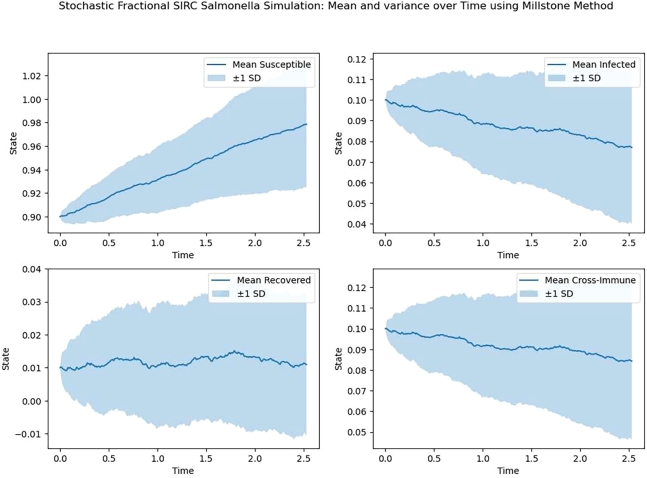
Mean and Variance for S, I, R, C.

In Table 2 our Milstein approximations of the model (2.2) were calculated for 10,000 sample paths for $N=2^{9},2^{10},2^{11},2^{12}$ and 2^13^ over [0,1] to estimate

$$E[Y(1)]\approx \frac{1}{10,000}\sum_{i=1}^{10,000}Y_{N}^{i},$$

where $Y_{N}^{i}$ is the estimate of *Y* at the end time T=1 for the I=1,2,3,4 the sample path using *N* subinterval. Table 3, Table 4, Table 5, Table 6, Table 7 show numerical results. ${\bar{X}}_{E}$ is the errors mean and confidence interval of errors in *k* iteration. Figure 2, Figure 3, Figure 4, Figure 5 also show the mean and confidence intervals for S, I, R, C.

**Table 3:** Estimation values for Milstein method.

***N***	S	I	R	C
2^9^	0.912648	0.093638	0.017230	0.067589
2^10^	0.934502	0.076814	0.015892	0.047047
2^11^	0.925521	0.097540	0.003762	0.067181
2^12^	0.914197	0.096625	0.005222	0.047002
2^13^	0.939128	0.089326	0.013408	0.063329

**Table 4:** Mean and confidence interval for S.

		95% Confidence Interval	
*t*_*i*_	${\bar{S}}_{E}$	Lower bound	Upper bound
0	0.9	0.9	0.9
0.1	0.89872	0.89865	0.8988
0.2	0.89749	0.8974	0.89759
0.3	0.89624	0.89613	0.89636
0.4	0.89505	0.89493	0.89518
0.5	0.89384	0.89369	0.89398
0.6	0.89259	0.89243	0.89274
0.7	0.8914	0.89123	0.89156
0.8	0.89025	0.89008	0.89043
0.9	0.88912	0.88893	0.88931
1	0.88796	0.88775	0.88816

**Table 5:** Mean and confidence interval for I.

		95% Confidence Interval	
*t*_*i*_	${\bar{I}}_{E}$	Lower bound	Upper bound
0	0.1	0.1	0.1
0.1	0.099265	0.099216	0.099314
0.2	0.098503	0.09843	0.098576
0.3	0.097748	0.097661	0.097836
0.4	0.097005	0.096908	0.097101
0.5	0.09629	0.096181	0.096399
0.6	0.0955	0.095375	0.095625
0.7	0.094757	0.094618	0.094895
0.8	0.094044	0.093894	0.094193
0.9	0.093341	0.093183	0.093499
1	0.092625	0.092462	0.092788

**Table 6:** Mean and confidence interval for R.

		95% Confidence Interval	
*t*_*i*_	${\bar{R}}_{E}$	Lower bound	Upper bound
0	0	0	0
0.1	0.00068759	0.00068658	0.0006886
0.2	0.0013184	0.0013169	0.0013199
0.3	0.0018979	0.0018958	0.0019001
0.4	0.0024291	0.002426	0.0024322
0.5	0.0029164	0.0029122	0.0029205
0.6	0.0033638	0.0033586	0.003369
0.7	0.0037727	0.0037666	0.0037788
0.8	0.0041467	0.0041396	0.0041537
0.9	0.0044901	0.0044821	0.0044981
1	0.0048037	0.0047946	0.0048128

**Table 7:** Mean and confidence interval for C.

		95% Confidence Interval	
*t*_*i*_	${\bar{C}}_{E}$	Lower bound	Upper bound
0	0	0	0
0.1	-8.2713e-05	-8.2851e-05	-8.2575e-05
0.2	-0.00020358	-0.00020388	-0.00020328
0.3	-0.00032149	-0.00032196	-0.00032102
0.4	-0.00042587	-0.00042642	-0.00042532
0.5	-0.00051278	-0.00051348	-0.00051207
0.6	-0.00058073	-0.00058157	-0.00057989
0.7	-0.0006293	-0.00063039	-0.00062821
0.8	-0.00065926	-0.00066058	-0.00065794
0.9	-0.00067181	-0.00067339	-0.00067024
1	-0.00066783	-0.00066962	-0.00066603

## 7 Properties of the deterministic model

The following norm will be defined: ${\left\|g(x)\right\|}_{\infty }=\sup_{x\in \varpi_{x}}|g(x)|$.

Theorem 4 *A solution of Salmonella bacteria infection model*(2.1)*in the fractional order derivative of a Caputo operator is singular and bound to*$R_{+}^{4}$*.*

Proof By following the same procedure as Fathalla et al. [51], it is possible to show that the Salmonella bacteria infection model (2.1) exists and is unique on the given initial (0,∞). Since

Dtυ0CS|S=0=μ+ηC≥0,Dtυ0CI|I=0=βIS+σβIC≥0,Dtυ0CR|R=0=β(1−σ)IC+θI≥0,Dtυ0CC|C=0=δR≥0.

If we have $\left(S(0),I(0),R(0),C(0)\right)\in R^{4}$, the solution of the model (2.1). (S,I,R,C) cannot outflow from the hyperplanes S=0,I=0,R=0,C=0. The hyperplane points towards the non-negative orthant $R_{+}^{4}$ and therefore, a positively invariant set. □

Theorem 5 *In the fractional-order model* (2.1) *, the solutions are uniformly bounded*

$$\Omega =\left\{(S,I,R,C)\in \Omega^{+}:0\leq N\leq \frac{\mu }{m}\right\}.$$

Proof Since

$$D_{t}N=\mu -(\mu +m)N\leq \mu -mN.$$

Thus

$$D_{t}N+mN\leq \mu .$$

Following to [56], by taking $M_{\upsilon }$ as the Mittag-Leffler function, one obtains

$$0\leq N\leq N(0)M_{\upsilon }(-mt^{\upsilon })+t^{\upsilon }M_{\upsilon ,\upsilon +1}(-mt^{\upsilon })).$$

According to [57], one obtains:

$$0\leq N\leq \frac{\mu }{m},\;t⟶\infty .$$

As a result, the solutions of (2.1), starting in $\Omega^{+}$, are uniformly bounded in the region Ω. □

The system of Salmonella bacteria infection (2.1) is can rewrite as:

(7.1) {Dtυ0Cϕ(t)=Ψ(ϕ(t)),0<t<T<∞,ϕ(0)=ϕ0.

The state vector ϕ(t)=(S(t),I(t),R(t),C(t)), while Ψ represents as:

$$\Psi =\left(\begin{array}{c} \Psi_{1}\\ \Psi_{2}\\ \Psi_{3}\\ \Psi_{4} \end{array}\right)=\left(\begin{array}{c} \mu +\eta C-(\mu +\beta I)S\\ \beta I\;S+\sigma \beta I\;C-(m+\mu +\theta )I\\ \beta (1-\sigma )I\;C+\theta I-(\delta +\mu )R\\ \delta R-\beta I\;C-(\mu +\eta )C. \end{array}\right).$$

In the Salmonella bacteria infection model (7.1), $\phi_{0}$ represents the initial state vector. As Ψ is a quadratic vector function, it fulfills the Lipschitz condition. Consequently, ∃ positive constant *η* satisfies

(7.2) ‖Ψ(ϕ)−Ψ(ψ)‖≤η‖ϕ−ψ‖.

Theorem 6 *In the model*(7.1)*, there is a distinct solution whenever*

(7.3) $$\frac{1}{\Gamma (\upsilon )}\eta \Gamma^{\upsilon }<1.$$

Proof Following Definition 2, one obtains:

(7.4) $$\phi =\phi_{0}+\frac{1}{\Gamma (\upsilon )}\int_{0}^{t}{\left(t-\epsilon \right)}^{\upsilon -1}\Psi (\phi (\epsilon ))d\epsilon .$$

Thus

(7.5) $$\Psi (\phi )=\phi_{0}+\frac{1}{\Gamma (\upsilon )}\int_{0}^{t}{\left(t-\epsilon \right)}^{\upsilon -1}\Psi (\phi (\epsilon ))d\epsilon .$$

Equation (7.4) is written as follows:

ϕ=Ψ(ϕ).

The supremum norm for *G* is ${\left\|\cdot \right\|}_{G}$.

$${\left\|\phi \right\|}_{G}=\underset{t\in G}{\sup}\|\phi \|,\;\phi \in E\left(G,R^{4}\right),$$

with Banach space $\left(E\left(G,R^{4}\right),{\left\|\cdot \right\|}_{G}\right)$. In addition, it can be demonstrated that

(7.6) $${\left\|\int_{0}^{t}K(t,\epsilon )\phi (\epsilon )d\epsilon \right\|}_{G}\leq T{\left\|K(t,\epsilon )\right\|}_{G}{\left\|\phi \right\|}_{G},$$

where $\phi \in E\left(G,R^{4}\right),K(t,\epsilon )\in E\left(G^{2},R\right)$ and

(7.7) $${\left\|K(t,\epsilon )\right\|}_{G},\underset{t,\epsilon \in G}{\sup}|K(t,\epsilon ),\;|K(t,\epsilon )\in E\left(G^{2},R\right).$$

Thus, applying (7.5), from (7.6) and (7.7), one obtains:

$${\left\|\Psi (\phi )-\Psi (\psi )\right\|}_{G}\leq \frac{1}{\Gamma (\upsilon )}T^{\upsilon }{\left\|G(\phi (\epsilon ))-G(\psi (\epsilon ))\right\|}_{G}\leq \left(\frac{1}{\Gamma (\upsilon )}\eta T^{\upsilon }\right){\left\|\phi -\psi \right\|}_{G}.$$

Thus, one obtains

$${\left\|\Psi (\phi )-\Psi (\psi )\right\|}_{G}\leq \eta {\left\|\phi -\psi \right\|}_{G},$$

where $L=\frac{1}{\Gamma (\upsilon )}\eta T^{\upsilon }$. Upon fulfillment of condition (7.3), the operator Ψ will be considered to be a contraction on $E\left(G^{2},R\right)$. As a result of Banach's fixed point theorem, model system (7.1) has a distinctive solution. □

Theorem 7 **[51]**

$E_{0}$ *is locally asymptotically stable and the infection will die out if* $R_{0}<1$ *and is unstable if* $R_{0}>1$ *.*

Proof $E_{0}$ is locally asymptotically stable if all the eigenvalues, $\lambda_{i},i=1,2,3$, 4, of Jacobian matrix $J\left(E_{0}\right)$ satisfy condition [58]:

$$\left|\arg\left(\lambda_{i}\right)\right|>\frac{\upsilon \pi }{2},$$

where

$$J\left(E_{0}\right)=\left(\begin{array}{cccc} -\mu \; & -\beta \; & 0\; & \eta \\ 0\; & \beta -(m+\mu +\theta )\; & 0\; & 0\\ 0\; & \theta \; & -(\delta +\mu )\; & 0\\ 0\; & 0\; & \delta \; & -(\mu +\eta ) \end{array}\right).$$

Thus $\lambda_{1}=-\mu ,\lambda_{2}=\beta -(m+\mu +\theta ),\lambda_{3}=$ $-(\delta +\mu ),\lambda_{4}=-(\mu +\eta )$. Hence, the proof follows.

Theorem 8 $E_{0}$*is globally asymptotically stable of model*(2.1)*if*$R_{0}<1$*and unstable if*$R_{0}>1$*.*

Proof Define a positive definite Lyapunov function:

$$L_{1}=(S-S_{0}-S_{0}\;\ln\frac{S}{S_{0}})+(R-R_{0}-R_{0}\;\ln\frac{R}{R_{0}}).$$

At $E_{0}$, one obtains

Dtυ0CL1≤(S−S0S)0CDtυS+(R−R0R)0CDtυR=(S−S0)(μS+ηCS−(βI+μ))+(R−R0)(δR−βCIR−(μ+η))=−μSS0(S−S0)2−ηCSS0(S−S0)2−δRRR0(R−R0)2−βCIRR0(R−R0)2.

Thus, Dtυ0CL1<0 for all (S,I,R,C)∈Ω. Moreover, Dtυ0CL1=0 implies that $S=S_{0}$, $I=I_{0}$, $R=R_{0}$, and $C=C_{0}$. So $\left\{E_{0}\right\}$ is the only set that satisfies Dtυ0CL1=0. □

At $E^{⋆}$, $J\left(E^{⋆}\right)$ is given by

$$J\left(E^{⋆}\right)=\left(\begin{array}{cccc} -\mu -\beta I^{⋆}\; & -\beta S^{⋆}\; & 0\; & \eta \\ \beta I^{⋆}\; & \beta S^{⋆}+\sigma \beta C^{⋆}-(m+\mu +\theta )\; & 0\; & \sigma \beta I^{⋆}\\ 0\; & (1-\sigma )\beta C^{⋆}+\theta \; & -(\delta +\mu )\; & (1-\sigma )\beta I^{⋆}\\ 0\; & -\beta C^{⋆}\; & \delta \; & -\beta I^{⋆}-(\mu +\eta ) \end{array}\right).$$

The characteristic polynomial is obtained as:

$$P(\varrho )=P=\det\left|I_{\varrho }-J\left(E^{⋆}\right)\right|=\varrho^{4}+{\overset{˜}{b}}_{1}\varrho^{3}+{\overset{˜}{b}}_{2}\varrho^{2}+{\overset{˜}{b}}_{3}\varrho +{\overset{˜}{b}}_{4}=0,$$

such that $I_{\varrho }$ is the 4×4 unit matrix with

$${\overset{˜}{b}}_{1}=\left(\varpi_{1}+\varpi_{3}+\varpi_{5}\right),{\overset{˜}{b}}_{2}=\left(\varpi_{1}\varpi_{3}-\varpi_{4}\delta +\varpi_{1}\varpi_{5}+\varpi_{3}\varpi_{5}+\beta^{2}I^{⋆}\;S^{⋆}+\sigma \beta^{2}C^{⋆}\;I^{⋆}\right),{\overset{˜}{b}}_{3}=(\varpi_{1}\varpi_{3}\varpi_{5}-\varpi_{1}\varpi_{4}\delta +\varpi_{3}\beta^{2}I^{⋆}\;S^{⋆}+\varpi_{5}\beta^{2}I^{⋆}\;S^{⋆}+\beta^{2}C^{⋆}\;\eta I^{⋆}\;\;-\varpi_{2}\sigma \beta \delta I^{⋆}+\sigma \beta^{2}\varpi_{1}C^{⋆}\;I^{⋆}+\sigma \varpi_{3}\beta^{2}C^{⋆}\;I^{⋆}),{\overset{˜}{b}}_{4}=\varpi_{3}\varpi_{5}\beta^{2}I^{⋆}\;S^{⋆}-\varpi_{2}\beta \delta \eta I^{⋆}+\varpi_{3}\beta^{2}C^{⋆}\;\eta I^{⋆}-\varpi_{4}\beta^{2}\delta I^{⋆}\;S^{⋆}-\sigma \beta \delta \varpi_{1}\varpi_{2}I^{⋆}+\sigma \varpi_{1}\varpi_{3}\beta^{2}C^{⋆}\;I^{⋆},\varpi_{1}=\beta i+\mu ,\;\varpi_{2}=\beta (1-\sigma )C^{⋆}+\theta ,\varpi_{3}=(\delta +\mu ),\;\varpi_{4}=\beta (1-\sigma )I^{⋆},\varpi_{5}=\beta I^{⋆}+(\mu +\eta ).$$

The discriminant of the polynomial: ϕ(μ)= $\varrho^{4}+{\overset{˜}{b}}_{1}\varrho^{3}+{\overset{˜}{b}}_{2}\varrho^{2}+{\overset{˜}{b}}_{3}\varrho +{\overset{˜}{b}}_{4}$ is given by

$$\overset{˜}{D}(\phi )=\left|\begin{array}{ccccccc} 1\; & {\overset{˜}{b}}_{1}\; & {\overset{˜}{b}}_{2}\; & {\overset{˜}{b}}_{3}\; & {\overset{˜}{b}}_{4}\; & 0\; & 0\\ 0\; & 1\; & {\overset{˜}{b}}_{1}\; & {\overset{˜}{b}}_{2}\; & {\overset{˜}{b}}_{3}\; & {\overset{˜}{b}}_{4}\; & 0\\ 0\; & 0\; & 1\; & {\overset{˜}{b}}_{1}\; & {\overset{˜}{b}}_{2}\; & {\overset{˜}{b}}_{3}\; & {\overset{˜}{b}}_{4}\\ 4\; & 3{\overset{˜}{b}}_{1}\; & 2{\overset{˜}{b}}_{2}\; & {\overset{˜}{b}}_{3}\; & 0\; & 0\; & 0\\ 0\; & 4\; & 3{\overset{˜}{b}}_{1}\; & 2{\overset{˜}{b}}_{2}\; & {\overset{˜}{b}}_{3}\; & 0\; & 0\\ 0\; & 0\; & 4\; & 3{\overset{˜}{b}}_{1}\; & 2{\overset{˜}{b}}_{2}\; & {\overset{˜}{b}}_{3}\; & 0\\ 0\; & 0\; & 0\; & 4\; & 3{\overset{˜}{b}}_{1}\; & 2{\overset{˜}{b}}_{2}\; & {\overset{˜}{b}}_{3} \end{array}\right|.$$

 □

Proposition 1 **[59]**

*Assume that* $E^{⋆}$ *exists in* $R_{+}^{4}$ *.*

(1) *Let*
  $${\overset{˜}{c}}_{1}={\overset{˜}{b}}_{1},\;{\overset{˜}{c}}_{2}=\left|\begin{array}{cc} {\overset{˜}{b}}_{1}\; & 1\\ {\overset{˜}{b}}_{3}\; & {\overset{˜}{b}}_{2} \end{array}\right|,\;{\overset{˜}{c}}_{3}=\left|\begin{array}{ccc} {\overset{˜}{b}}_{1}\; & 1\; & 0\\ {\overset{˜}{b}}_{3}\; & {\overset{˜}{b}}_{2}\; & {\overset{˜}{b}}_{1}\\ 0\; & {\overset{˜}{b}}_{4}\; & {\overset{˜}{b}}_{3} \end{array}\right|,$$
  *be the Routh-Hurwitz determinants. Therefore, when* υ=1 *,* $E^{⋆}$ *is locally asymptotically stable if*
  (7.8) $${\overset{˜}{c}}_{1}>0,\;{\overset{˜}{c}}_{2}>0,\;{\overset{˜}{c}}_{3}=0,\;{\overset{˜}{b}}_{4}>0.$$
  $E^{⋆}$ *is locally asymptotically stable for all* υ∈[0,1] *, whenever* (7.8) *are sufficient (not necessary).*
(2) *If* $\overset{˜}{D}(\phi )>0,{\overset{˜}{b}}_{1}>0,{\overset{˜}{b}}_{2}<0$ *, and* υ>2/3 *, then* $E^{⋆}$ *is unstable.*
(3) *If*$\overset{˜}{D}(\phi )<0,{\overset{˜}{b}}_{1}>0,{\overset{˜}{b}}_{2}>0,{\overset{˜}{b}}_{3}>0,{\overset{˜}{b}}_{4}>0$*, and υ*< 1/3*, then* $E^{⋆}$ *is locally asymptotically stable. Also, if* $\overset{˜}{D}(\phi )<0,{\overset{˜}{b}}_{1}<0,{\overset{˜}{b}}_{2}>0,{\overset{˜}{b}}_{3}<0$*,* ${\overset{˜}{b}}_{4}>0$*,* $E^{⋆}$ *is unstable.*
(4) *If* $\overset{˜}{D}(\phi )<0,{\overset{˜}{b}}_{1}>0,{\overset{˜}{b}}_{2}>0,{\overset{˜}{b}}_{3}>0,{\overset{˜}{b}}_{4}>0$ *, and* ${\overset{˜}{b}}_{2}=\left({\overset{˜}{b}}_{1}{\overset{˜}{b}}_{4}/{\overset{˜}{b}}_{3}\right)+\left({\overset{˜}{b}}_{3}/{\overset{˜}{b}}_{1}\right)$ *,* $E^{⋆}$ *is locally asymptotically stable for all* υ∈(0,1) *.*
(5) $E^{⋆}$ *is locally asymptotically stable if* ${\overset{˜}{b}}_{4}>0$ *.*

Lemma 1 $E^{⁎}$ *is locally asymptotically stable for* $R_{0}>1$ *. Moreover,* $E^{⁎}$ *is globally asymptotically stable.*

Proof Define

$$\begin{array}{cc} L_{2} & =(S-S^{⋆}-S^{⋆}\;\ln\frac{S}{S^{⋆}})+(I-I^{⋆}-I^{⋆}\;\ln\frac{I}{I^{⋆}})\\ & +(R-R^{⋆}-R^{⋆}\;\ln\frac{R}{R^{⋆}})+(C-C^{⋆}-C^{⋆}\;\ln\frac{C}{C^{⋆}}). \end{array}$$

Thus, one obtains

$$\begin{array}{cc} D_{t}^{\upsilon }L_{2} & \leq (\frac{S-S^{⋆}}{S}){}_{0}^{C}D_{t}^{\upsilon }S+(\frac{I-I^{⋆}}{I}){}_{0}^{C}D_{t}^{\upsilon }I+(\frac{R-R^{⋆}}{R}){}_{0}^{C}D_{t}^{\upsilon }R+(\frac{C-C^{⋆}}{C}){}_{0}^{C}D_{t}^{\upsilon }C\\ & =-\frac{\mu }{SS^{⋆}}{\left(S-S^{⋆}\right)}^{2}-\frac{\eta C}{SS^{⋆}}{\left(S-S^{⋆}\right)}^{2}-\frac{\beta (1-\sigma )C\;I}{RR^{⋆}}{\left(R-R^{⋆}\right)}^{2}\\ & -\frac{\theta I}{RR^{⋆}}{\left(R-R^{⋆}\right)}^{2}-\frac{\delta R}{CC^{⋆}}{\left(C-C^{⋆}\right)}^{2}. \end{array}$$

Thus, Dtυ0CL2<0 for all (S,I,R,C)∈μ. Furthermore Dtυ0CL2=0 implies that $S=S^{⋆}$, $I=I^{⋆}$, $R=R^{⋆}$, $C=C^{⋆}$. Therefore, as Theorem 8, $E^{⋆}$ is globally asymptotically stable. □

Definition 8 **[60], [61]**

The constants $\zeta_{i}>0$, for $i\in N_{1}^{4}$ must meet the following conditions for every $\zeta_{i}>0$, $i\in N_{1}^{4}$, for model (2.1) to be Hyers-Ulam Stability:

$$|S(t)-\frac{1}{\Gamma (\upsilon )}\int_{0}^{t}W^{\theta_{2}-1}\;{\left(t-W\right)}^{\theta_{1}-1}{ϒ}_{1}(W,\Psi (W))d\;W|\leq \zeta_{1},$$

$$|I(t)-\frac{1}{\Gamma (\upsilon )}\int_{0}^{t}W^{\theta_{2}-1}\;{\left(t-W\right)}^{\theta_{1}-1}{ϒ}_{2}(W,\Psi (W))d\;W|\leq \zeta_{2},$$

$$|R(t)-\frac{1}{\Gamma (\upsilon )}\int_{0}^{t}W^{\theta_{2}-1}\;{\left(t-W\right)}^{\theta_{1}-1}{ϒ}_{3}(W,\Psi (W))d\;W|\leq \zeta_{3},$$

$$|C(t)-\frac{1}{\Gamma (\upsilon )}\int_{0}^{t}W^{\theta_{2}-1}\;{\left(t-W\right)}^{\theta_{1}-1}{ϒ}_{4}(W,\Psi (W))d\;W|\leq \zeta_{4}.$$

In the model (2.1), an approximation is $\left(S_{1}(t),I_{1}(t),R_{1}(t),C_{1}(t)\right)$ that meets:

$$S_{1}(t)=\frac{1}{\Gamma (\upsilon )}\int_{0}^{t}W^{\theta_{2}-1}\;{\left(t-W\right)}^{\theta_{1}-1}{ϒ}_{1}(W,S_{1}(W))d\;W,$$

$$I_{1}(t)=\frac{1}{\Gamma (\upsilon )}\int_{0}^{t}W^{\theta_{2}-1}\;{\left(t-W\right)}^{\theta_{1}-1}{ϒ}_{2}(W,I_{1}(W))d\;W,$$

$$R_{1}(t)=\frac{1}{\Gamma (\upsilon )}\int_{0}^{t}W^{\theta_{2}-1}\;{\left(t-W\right)}^{\theta_{1}-1}{ϒ}_{3}(W,R_{1}(W))d\;W,$$

$$C_{1}(t)=\frac{1}{\Gamma (\upsilon )}\int_{0}^{t}W^{\theta_{2}-1}\;{\left(t-W\right)}^{\theta_{1}-1}{ϒ}_{4}(W,C_{1}(W))d\;W,$$

so that

(7.9) $$|S-S_{1}|\leq v_{1}\;\omega_{1},|I-I_{1}|\leq v_{2}\;\omega_{2},|R-R_{1}|\leq v_{3}\;\omega_{3},|C-C_{1}|\leq v_{4}\;\omega_{4}.$$

Theorem 9 *The model* (2.1) *is Hyers-Ulam Stability, if* (7.9) *is true.*

Proof $$|S-S_{1}|=\frac{\theta_{1}\theta_{2}}{\hbar (\theta_{1})\Gamma \left(\theta_{1}\right)}\int_{0}^{t}W^{\theta_{2}-1}\;{\left(t-W\right)}^{\theta_{1}-1}({ϒ}_{1}(W,S(W))-{ϒ}_{1}(W,S_{1}(W)))d\;W|\leq \frac{\theta_{1}\theta_{2}}{\hbar (\theta_{1})\Gamma \left(\theta_{1}\right)}\int_{0}^{t}W^{\theta_{2}-1}\;{\left(t-W\right)}^{\theta_{1}-1}\omega_{1}\left\|S-S_{1}\right\|d\;W\leq \frac{1}{\Gamma (\upsilon )}\omega_{1}\left\|S-S_{1}\right\|\text{. }$$

Then

$$|S-S_{1}|\leq v_{1}\;\omega_{1},\text{ with }v_{1}=\frac{1}{\Gamma (\upsilon )}\left\|S-S_{1}\right\|.$$

Similarly, one obtains

$$|I-I_{1}|\leq v_{2}\;\omega_{2},\text{ with }v_{2}=\frac{1}{\Gamma (\upsilon )}\left\|I-I_{1}\right\|,|R-R_{1}|\leq v_{3}\;\omega_{3},\text{ with }v_{3}=\frac{1}{\Gamma (\upsilon )}\left\|R-R_{1}\right\|,|C-C_{1}|\leq v_{4}\;\omega_{4},\text{ with }v_{4}=\frac{1}{\Gamma (\upsilon )}\left\|C-C_{1}\right\|.$$

Hence the results follow. □

## 8 Comparison of stochastic and deterministic fractional SIRC models

**Figure 6:**
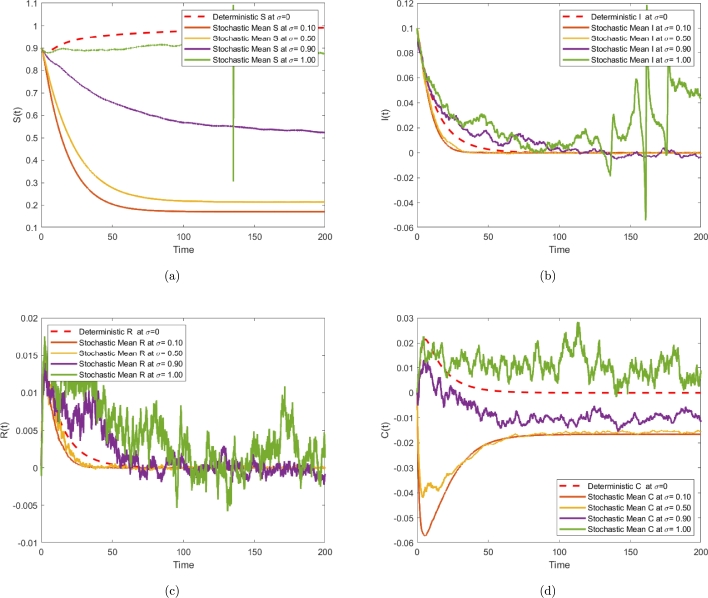
Comparison of Stochastic and Deterministic Fractional SIRC Models with Different Noise Levels.

The Milstein numerical approximation method is used in [18] to study the dynamics of the model (2.2). This is done by comparing the stochastic system (2.2) to its deterministic counterpart (2.1), with $\sigma_{1}=0.1,\sigma_{1}=0.5,\sigma_{1}=0.9,\sigma_{1}=1$ noise value applied to the stochastic system (2.2). As a result, the computer running results presented in Fig. 6 indicate that the inclusion of random noise in the system leads to extinction and reductions of both infected individuals (with bacterial infection), as well as cross-immune individuals. It illustrates the effect of noise intensity on bacterial spread. In this case, a large noise value controlled the epidemic propagation (see Fig. 6).

**Figure 7:**
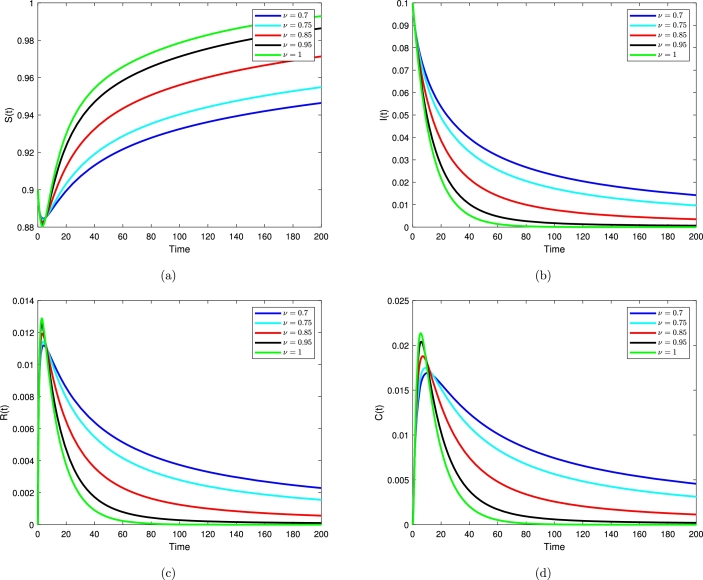
Time series plot for S, I, R, C.

**Figure 8:**
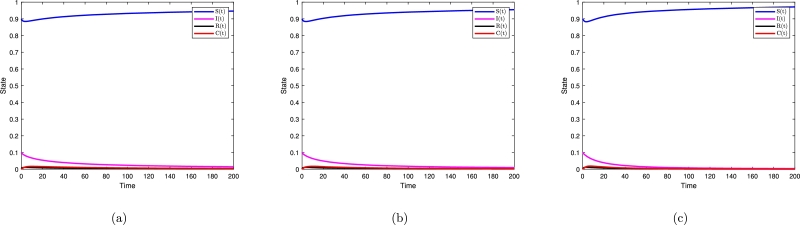
Dynamics of system (2.2) for (a) *υ* = 0.75, *υ* = 0.95, (b) *υ* = 1.

Fig. 6 shows that the Milstein numerical approximation method to compare the models (2.1) and (2.2). Figs. 7 show the behavior of the deterministic Salmonella bacteria infection model transmission, υ=0.7, υ=0.75, υ=0.85, υ=0.95, υ=1. The dynamic of deterministic differential system, can also be seen in Figs. 8 for various υ=0.7, υ=0.95, υ=1. A stochastic fractional model of order 0.95 gives the highest expected peak value of daily exposed individuals. Summary, although deterministic models offer simplicity and computational efficiency, stochastic fractional SIRC models provide a more nuanced insight into the dynamics of diseases as compared to deterministic models. Particularly in those situations where randomness and variability play a significant role, this is especially true. This allows one to determine what model is suitable for a specific research question, what data are available for the study, and how much computational power to devote to it.

## 9 Discussion

This study aims to provide a comprehensive understanding of Salmonella infection dynamics, combining theoretical modeling with numerical simulations to uncover key insights into the behavior of the system under stochastic influences and environmental factors. In this paper, covering various aspects of Salmonella infection dynamics through mathematical modeling. Using stochastic differential equations perturbed with white noise and implementing the Milstein scheme demonstrates a sophisticated approach to capturing the stochastic nature of the system. Comparing the Milstein scheme's results with different discretization values and sample paths provides valuable insights into its efficiency and accuracy. The Milstein scheme, derived from a truncated Ito-Taylor expansion, offers a practical method for numerically approximating the solutions of these stochastic models. Its efficiency lies in its ability to accurately capture both the deterministic trend and the stochastic fluctuations in the system. By implementing the Milstein scheme with different discretization values and sample paths, we've systematically evaluated its performance and demonstrated its effectiveness in capturing the dynamics of Salmonella infections. It's promising to see that the numerical solutions converge towards the approximate solution of the deterministic model as the discretization value increases, validating the effectiveness of the Milstein method. Moreover, this study highlights the importance of incorporating stochasticity into epidemic models, as evidenced by the comparison between models (2.1) and (2.2). While deterministic models provide valuable insights into the average behavior of the system, they often overlook the inherent randomness present in real-world scenarios. By introducing stochastic elements, such as environmental variability and individual-level heterogeneity, the proposed stochastic model more accurately reflects the unpredictable nature of infectious disease spread. The observed reduction in Salmonella infections due to randomness underscores the critical role of stochastic processes in shaping epidemic dynamics. It's intriguing that environmental regimes play a distinct role in shaping epidemic model parameters, emphasizing the importance of considering real-world conditions in modeling infectious diseases. Additionally, the derivation of a fractional order modified SIRC model via Euler's method and the numerical solution of the remaining stochastic models using the Milstein method with specific parameters demonstrate the versatility in employing different numerical techniques to tackle complex systems. Investigating the distinct effects of different environmental regimes on epidemic model parameters adds a nuanced layer to this study. Environmental factors, such as temperature, humidity, and population density, can significantly influence pathogen transmission rates and disease dynamics. By incorporating these factors into the proposed models, we've demonstrated how variations in environmental conditions can lead to different disease outcomes. This analysis not only enhances our understanding of Salmonella infection dynamics but also underscores the importance of considering real-world complexities in epidemiological modeling. The numerical methods used for model solution showcases a combination of numerical techniques, including Euler's method for fractional order modified SIRC model and the Milstein method for stochastic models. Each method offers unique advantages and challenges, depending on the specific characteristics of the model and the desired level of accuracy. By carefully selecting and implementing these numerical approaches, we've successfully obtained solutions that capture the intricate dynamics of Salmonella infections. Additionally, the comparison of curve peaks and action times between deterministic and stochastic models provides valuable insights into how stochasticity affects disease progression and intervention strategies.

## 10 Conclusion

To study Salmonella infection dynamics, we developed a stochastic differential equation mathematical model. Susceptible, infected, recovered, and cross-immune herds are all considered in the proposed model. The model (2.2) has been perturbed with white noise. A truncated Ito-Taylor expansion has already been proposed in [52] leading to a Milstein scheme. For illustrating Milstein's method's efficiency, we implemented the scheme into a nonlinear stochastic differential equation. Moreover, we investigated approximations for $2^{9},2^{10},2^{11},2^{12}$ and 2^13^ discretization in the interval [0,1] with 10000 different sample paths. In our study, we found that the numerical solution obtained from the Milstein scheme was close to the approximate solution of the deterministic model as the discretization value *N* increased, and the results in the tables indicate that the Milstein method is effective. Using the Milstein numerical approximation method, we compared models (2.1) and (2.2). Based on the data in [55], we created graphs that indicate that randomness reduces Salmonella infections. Every environmental regime has a distinct effect on epidemic model parameters. The fractional order modified SEIRF model is derived via Euler's method with step time set to 0.001 days. The remaining two stochastic models are solved numerically using the Milstein method with number of paths equals 10 and discretization of time equals 0.001 day. The average path solution is taken. Stochastic models give different curve peaks but also different action times.

## CRediT authorship contribution statement

**Salem Mubarak Alzahrani:** Writing – review & editing, Writing – original draft, Visualization, Validation, Supervision, Software, Resources, Project administration, Methodology, Investigation, Funding acquisition, Formal analysis, Data curation, Conceptualization.

## Declaration of Competing Interest

The authors declare that they have no known competing financial interests or personal relationships that could have appeared to influence the work.

## Data Availability

Data included in the article or referenced in the article.
